# Secondary infection in COVID-19 critically ill patients: a retrospective single-center evaluation

**DOI:** 10.1186/s12879-022-07192-x

**Published:** 2022-03-02

**Authors:** Astrid De Bruyn, Stijn Verellen, Liesbeth Bruckers, Laurien Geebelen, Ina Callebaut, Ilse De Pauw, Björn Stessel, Jasperina Dubois

**Affiliations:** 1 Department of Intensive Care and Anesthesiology, Jessa Hospital – Hasselt, 3500 Hasselt, Belgium; 2 I-BioStat, Data Science Institute, Hasselt University, Martelarenlaan 42, 3500 Hasselt, Belgium; 3 UHasselt, Faculty of Medicine and Life Sciences, LCRC, Agoralaan, 3590 Diepenbeek, Belgium

**Keywords:** COVID-19, Bacterial infection, Intensive Care Unit

## Abstract

**Background:**

Patients infected with severe acute respiratory syndrome coronavirus (SARS-CoV-2) can develop severe illness necessitating intensive care admission. Critically ill patients are susceptible for the development of secondary bacterial infections. Due to a combination of virus- and drug-induced immunosuppression, critically ill patients with corona virus disease 2019 (COVID-19) may even have a higher risk of developing a secondary infection. These secondary infections can aggravate the severity of illness and increase the risk of death. Further research on secondary infections in COVID-19 patients is essential. Therefore, the objective of this study was to investigate the incidence and associated risk factors of secondary bacterial infections and to identify the most common groups of pathogens in critically ill COVID-19 patients.

**Methods:**

This mono-center, retrospective observational cohort study was performed at the intensive care unit (ICU) of the Jessa Hospital, Hasselt, Belgium. All adult COVID-19 patients admitted to the ICU from 13th March 2020 until 17th October 2020, were eligible for inclusion in the study. Data from the resulting 116 patients were prospectively entered into a customized database. The resulting database was retrospectively reviewed to investigate three types of secondary bacterial infections (secondary pneumonia, bloodstream infections of unknown origin, catheter-related sepsis).

**Results:**

Of 94 included patients, 68% acquired at least one of the studied secondary bacterial infections during their ICU stay. Almost two thirds of patients (65.96%, n = 62) acquired a secondary pneumonia, whereas 29.79% (n = 28) acquired a bacteremia of unknown origin and a smaller proportion of patients (14.89%, n = 14) acquired a catheter-related sepsis. Male gender (P = 0.05), diabetes mellitus (P = 0.03) and the cumulative dose of corticosteroids (P = 0.004) were associated with increased risk of secondary bacterial infection. The most common pathogens detected in the cultures of patients with secondary pneumonia were Gram-negative bacilli. Bacteremia of unknown origin and catheter-related sepsis were mostly caused by Gram-positive cocci.

**Conclusion:**

This study confirms that the incidence of secondary bacterial infections is very high in critically ill COVID-19 patients. These patients are at highest risk of developing secondary pneumonia. Male gender, a history of diabetes mellitus and the administration of corticosteroids were associated with increased risk of secondary bacterial infection.

**Supplementary Information:**

The online version contains supplementary material available at 10.1186/s12879-022-07192-x.

## Background

Severe acute respiratory syndrome coronavirus 2 (SARS-CoV-2) is the cause of the ongoing pandemic of coronavirus disease (COVID-19). The spectrum of disease severity of patients infected with SARS-CoV-2 is very wide: from an asymptomatic carrier state to severe lower respiratory tract infection and critical illness with intensive care unit (ICU) requirement [1].

In general, critically ill patients are susceptible for development of secondary bacterial infections such as secondary pneumonia, bloodstream infection of unknown origin, and-catheter related sepsis. It has been suggested that critically ill patients infected with SARS-CoV-2 are even at higher risk of developing secondary infections due to a combination of virus- and drug-induced immunosuppression [2]. Secondary infections may lead to a lower discharge rate and higher mortality rate [3]. As a result, further research on secondary infections in COVID-19 patients is essential.

In literature, there is a wide variation in reported incidences and outcomes of secondary bacterial infections in critical ill COVID-19 patients. A recent systematic review reported an incidence of secondary infections ranging from 7% up to 51% in critically ill patients infected with SARS-CoV-2 [4]. The most common bacterial complication of COVID-19 was secondary pneumonia including ventilator-assisted pneumonia (VAP). A recent multicenter study described a cumulative incidence of VAP of 50% in patients with COVID-19 admitted to the ICU [5]. Data on bloodstream infections in critically ill COVID-19 patients are rather scarce. It has been suggested that bloodstream infections are the second most common secondary infection in critically ill COVID-19 patients with incidences ranging from 3.4 to 50% [6, 7].

Identification of the pathogens most often responsible for the development of secondary infections in critical ill COVID-19 patients generates new possibilities such as individually tailored empiric antibiotic therapy in those patients with early signs of a secondary infection. Furthermore, identification of possible risk factors associated with secondary bacterial infections may lead to development of new prevention strategies for secondary infections.

Therefore, the objective of this study was to investigate the incidence and possible risk factors of secondary bacterial infections and to identify the most common groups of pathogens in critically ill patients infected with SARS-CoV-2.

## Methods

### Patients and study design

This mono-center, investigator-initiated, longitudinal, retrospective observational cohort study was performed at the ICU of the Jessa Hospital, Hasselt, Belgium, after approval by the ethical committee of Jessa Hospital, Hasselt, Belgium on 14th April 2021 (2021-037) and registration on clinicaltrials.gov (NCT04877808). Written informed consent was waived considering the urgent need to collect data on the ongoing pandemic and the retrospective nature of this study. This study is reported according to the STrengthening the Reporting of OBservational studies in Epidemiology (STROBE) statement [8].

All adult patients, with acute hypoxemic respiratory failure due to diagnosed COVID-19 pneumonia admitted to the ICU of Jessa from 13th March 2020 until 17th October 2020, were eligible for inclusion in the study. In accordance with the World Health Organisation (WHO) protocol [9], laboratory confirmation of COVID-19 infection was defined as a positive result on polymerase chain reaction (PCR) assays of nasopharyngeal swab samples or on bronchoalveolar lavage. This resulted in the screening of 116 COVID-19 patients admitted to the ICU. All patients were treated according to the COVID-protocol of the Jessa hospital (Additional file 1: Appendix), based on the latest insights on COVID-19 at that time point [10]. Data from all patients were prospectively entered into a customized database that included medical history, demographic data, clinical parameters, and laboratory results. Scores of severity of illness, such as APACHE IV scores and Sequential Organ Failure Assessment (SOFA) score, were calculated on ICU admission. The following outcome measures were investigated: ICU mortality, need of mechanical ventilation and length of stay (LOS) in the ICU and hospital LOS. Following completion of the database, the data were retrospectively reviewed.

### Diagnosis of secondary bacterial infection

The diagnosis of a secondary bacterial infection was based on clinical symptoms in combination with laboratory analyses. Secondary pneumonia was diagnosed when the patient developed clinical symptoms, positive radiologic signs and had positive laboratory-confirmed culture from the lower respiratory tract [4, 5]. The diagnosis of blood stream infection of unknown origin was based on clinical symptoms in combination with a positive blood culture, in the absence of a confirmed or suspected origin of the infection [6, 7]. Catheter-related sepsis was diagnosed in the case of clinical symptoms related to sepsis in combination with a positive culture originating from a catheter [7]. All positive cultures were further analyzed to identify the responsible pathogen.

### Statistical analyses

Continuous data are shown as mean ± standard deviation (SD) and categorical data are presented as frequencies and percentages. Comparisons between groups were performed with the Student’s *t*-tests for normally distributed data and with Mann Whitney U test for not normally distributed data. Categorical variables were analyzed with a Chi-Square test or, if appropriate, with a Fisher’s exact test. Univariable logistic regression was used to investigate the association of possible risk factors (age, gender, BMI, diabetes mellitus, hypertension, smoking, APACHE IV score, SOFA score on admission, need for hemodynamic assist device, cumulative dose of corticosteroids administered in the ICU, and other immune suppressive therapies) and the acquisition of a secondary bacterial infection. A P-value < 0.05 was considered statistically significant.

## Results

**Fig. 1:**
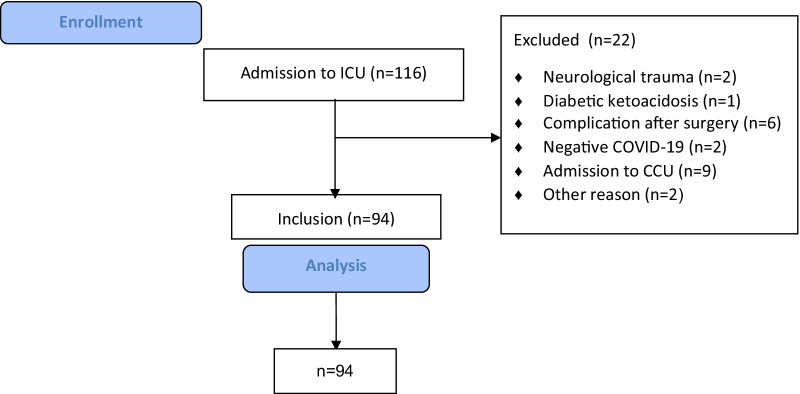
CONSORT flow chart depicting exclusion and inclusion of patients

A STROBE flow chart depicting exclusion and inclusion of patients is shown in Fig. 1

In total, 22 patients were excluded. This resulted in data of 94 patients for final analysis.

### Baseline characteristics

Baseline characteristics stratified for the occurrence of secondary infections are presented in Table 1.

**Table 1:** Baseline characteristics

Total study cohort n = 94	No secondary infection n = 30	Secondary infection n = 64	P value
*Baseline characteristics*			
Age (years)	71.43 ± 9.65	67.92 ± 10.86	0.13
BMI (kg/m^2^)	26.99 ± 6.68	28.09 ± 5.63	0.99
Gender (male)	13 (43.3%)	42 (65.6%)	**0.04**
DNI code	9 (30.0%)	6 (9.4%)	**0.01**
No DNI code	21 (70%)	58 (90.6%)	
*Co-morbidities*			
Smoking	1 (3.3%)	3(4.7%)	0.75
Reumatological disease	4 (13.3%)	4 (6.3%)	0.25
Obesity	9 (30.0%)	22 (34.4%)	0.67
Arterial hypertension	22 (73.3%)	36 (56.3%)	0.11
Diabetes mellitus	14 (46.7%)	18 (28.1%)	0.07
*Severity of illness* PaO_2_/FiO_2_ at admission Worst PaO_2_/FiO_2_	126.5 ± 108.5 103.4 ± 90.16	149.0 ± 158.6 91.26 ± 60.91	0.44 0.73
APACHE IV score	50.1 ± 16.7	47.0 ± 15.9	0.97
SOFA score	5.2 ± 3.4	4.5 ± 3.2	0.20

Data are presented as mean ± SD or frequencies (%). A P-value < 0.05 is considered statistically significant

*p < 0.05

### Outcome measures

Outcome measures of the study population are presented in Table 2. There is a significant longer ICU stay (P < 0.001), longer hospital stay (P < 0.001), and longer need of mechanical ventilation (P < 0.001) in the cohort of patients diagnosed with a secondary bacterial infection.

**Table 2:** Outcome measures

Total study cohort n = 94	No secondary infection n = 30	Secondary infection n = 64	P value
*Outcome measures*			
LOS ICU (days)	5.93 ± 2.66	25.00 ± 19.46	< 0.001
DNI	5.11 ± 1.17	15.50 ± 6.12	
Non DNI	6.28 ± 3.05	25.98 ± 20.13	
LOS hospital (days)	13.56 ± 6.83	38.08 ± 26.40	< 0.001
DNI	12.33 ± 8.29	27.67 ± 7.89	
Non DNI	14.10 ± 6.26	39.16 ± 27.42	
ICU mortality	12 (40.0%)	17 (26.6%)	0.18
DNI	8 (66.6%)	3 (17.6%)	
Non DNI	4 (8.4%)	14 (82.4%)	
Mechanical ventilation	6 (20.0%)	46 (71.9%)	< 0.001

Data are presented as mean ± SD or frequencies (%). A P-value < 0.05 is considered statistically significant

### Incidence of secondary bacterial infection

**Fig. 2:**
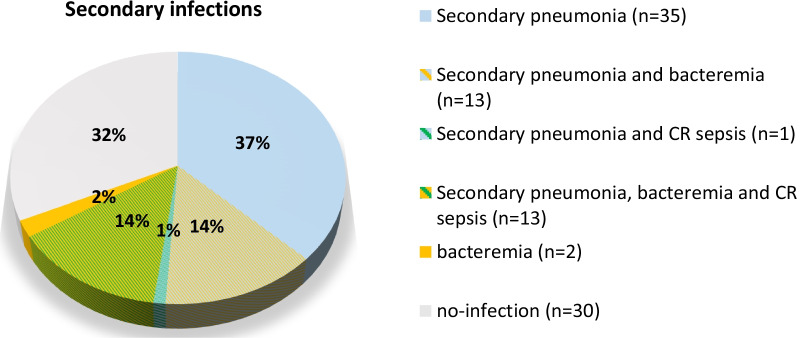
Incidence of secondary infections

The incidence of all three different types of diagnosed secondary bacterial infections is visualized in Fig. 2. More than two third of included patients (n = 64, 68.1%) suffered from at least one of three predefined secondary bacterial infections (secondary pneumonia, bloodstream infection of unknown origin, catheter-related sepsis). Secondary pneumonia was the most frequently diagnosed secondary infection (n = 61, 64.9%). In 35 (57.4%) patients diagnosed with secondary pneumonia, the lung was the only secondary infection site. Blood stream infection of unknown origin was diagnosed in 28 (29.8%) patients. A smaller proportion of patients (14.9%, n = 14) acquired a catheter-related sepsis. Only 30 patients (31.9%) did not acquire one of the previous described secondary infections and 27 (28.7%) patients acquired more than one secondary infection.

### Risk factors associated with the acquisition of a secondary infection

Univariate regression analyses showed that only male gender (P = 0.05), diabetes mellitus (P = 0.03), and the treatment with corticosteroids were associated with a higher likelihood of acquiring a secondary bacterial infection (P = 0.004) (Table 3).

**Table 3:** Risk factors for the acquisition of secondary infection

Risk factors	n = 94	Odds ratio (95% CI)	P value
*Demographics*			
Age (years)—median [IQR]^2^	70 (62–77)	0.96 (0.92–1.01)	0.13
Male gender—no. (%)	**55 (59%)**	**0.41 (0.17–0.99)**	**0.05**
BMI—median [IQR]^2^	27.5 (24.2–30.8)	1.007 (0.926–1.095)	0.87
Diabetes mellitus—no. (%)	**32 (34%)**	**0.37 (0.15–0.91)**	**0.03**
Hypertension—no. (%)	58 (62%)	0.48 (0.19–1.24)	0.13
Smoking—no. (%)	4 (4%)	1.12 (0.13–9.93)	0.92
*Characteristics of type and severity of illness*			
APACHE IV score—median [IQR]^2^	46 (35–57)	0.99 (0.96–1.02)	0.35
SOFA score on admission—median [IQR]^2^	3 (2–5)	1.14 (0.96–1.35)	0.13
Hemodynamic assist device—no. (%)	4 (4%)	4.54 (0.17–122.37)	0.37
*Treatment*			
Corticosteroids dose—median [IQR]^2^	**0 (0–384)**	**1.004 (1.001–1.007)**	**0.004**
Other immunosuppressive therapies*—no. (%)	7 (7%)	0.59 (0.12–2.78)	0.50

*Other immunosuppressive therapies included: infliximab, ruxolitinib, bortezomib, mycophenolate mofetil, azacitidine and azathioprine

*p < 0.05

### Identification of most frequent pathogens

The identification of different types of pathogenic germs detected in the samples of the different infection sites are listed in Table 4. The most frequent types of pathogens identified in the cultures of patients with secondary pneumonia were Gram-negative bacilli (82%) followed by Gram-positive cocci (66%), Gram-negative cocci (24%), and Gram-positive bacilli (19%). Furthermore, in 12 patients (19.7%) with a secondary pneumonia, aspergillosis was detected in the cultures from the lower respiratory tractus. The most prevalent pathogens in bloodstream infection of unknown origin were Gram-positive cocci (89%) followed by Gram-negative bacilli (32%), Gram-negative cocci (18%), and Gram-positive bacilli (4%). In catheter related sepsis, Gram-positive cocci (79%) were the most prevalent whereas Gram-negative bacilli (36%), Gram-negative cocci (14%) and Gram-positive bacilli (7%) were less common.

**Table 4:** Bacteria responsible for secondary infection

Type of bacteria	Secondary pneumonia (n = 62)	Bacteremia (n = 28)	CR-sepsis (n = 14)
Gram-positive coccus	41 (66%)	25 (89%)	11 (79%)
Gram-negative coccus	15 (24%)	5 (18%)	2 (14%)
Gram-positive bacillus	12 (19%)	1 (4%)	1 (7%)
Gram-negative bacillus	51 (82%)	9 (32%)	5 (36%)

## Discussion

In this observational cohort study, we observed a very high incidence of 68% of secondary infections in critically ill patients infected with SARS-CoV-2. Secondary pneumonia (65%) was the most frequently diagnosed secondary infection, followed by bloodstream infection of unknown origin (30%) and catheter-related sepsis (15%). This study suggests that patients suffering from secondary infections may be at higher risk of longer ICU and hospital stay but not of ICU death.

Our results on the high incidence of secondary pneumonia are congruent with other cohort studies investigating secondary pneumonia in critically ill patients infected with SARS-CoV-2 [11]. Remarkably, the observed incidence is threefold higher than the incidence of secondary pneumonia in critically ill patients not infected with SARS-CoV-2 (13.5–23%) [12]. Other respiratory viral infections, such as seasonal/pandemic influenza, Middle East respiratory syndrome coronavirus and SARS-CoV-1 are also characterized by a high incidence of secondary pneumonia [13–15]. Bacterial infections responsible for secondary pneumonia originate from co-pathogens that can be found in the respiratory tract. In literature, it has been suggested that this type of pneumonia in critically ill patients may aggravate severity of illness and increases the risk of death [13–17]. In this study however, we couldn’t detect an increased risk of death in patients suffering from a secondary bacterial infection. It can be deduced from Table 2 that patients with a DNI code had a large impact on ICU mortality and other outcome variables. This impact most likely explains why we couldn’t detect an association between ICU mortality and acquisition of a secondary infection.

In this study cohort the most common type of pathogens responsible for secondary pneumonia were the Gram-negative bacilli, followed by Gram-positive cocci. This observation is consistent with what we would expect based on literature. A large multicenter study, performed in 36 European ICUs, identified Gram-negative bacilli, mainly *Pseudomonas auruginosa*, *Enterobacter* species, and *Escherichia coli* as the most common bacteria involved in secondary pneumonia in COVID-19 patients [5].

Besides secondary bacterial pneumonia, patients with a viral pneumonia in general, are prone for the development of invasive pulmonary aspergillosis, which has a negative impact on duration of hospitalization and mortality [18]. There is some data available, that suggest that critically ill COVID-19 patients have an even higher risk to develop invasive aspergillosis [19]. In this study cohort, the incidence of invasive aspergillosis in patients with a secondary pneumonia was 19.7%. This is in line with other European studies, which report a rate of 20–35% for invasive aspergillosis in critically ill COVID-19 patients with a secondary pneumonia [20, 21].

The incidence of blood stream infection and catheter-related sepsis was comparable with the incidence of secondary infection in critically ill patients not infected with SARS-CoV-2 [22]. The most common pathogens identified in blood cultures or catheter cultures from patients with a bloodstream infection or catheter-related sepsis were Gram-positive cocci. This is in line with the fact that *Staphylococcus aureus* is frequent a commensal on the skin and often responsible for blood stream infections and catheter-related infections.

The only identified risk factors associated with the acquisition of a secondary infection were a history of diabetes mellitus, the cumulative dose of corticosteroids administered in the ICU, and male gender. However, the investigated cohort was relatively small, thus it is presumable that other risk factors associated with the acquisition of a secondary infection could not be detected. The first identified risk factor, diabetes mellitus, is known to increase the incidence and severity of infectious diseases. Previous research indicates that diabetic patients with COVID-19 admitted to an ICU also have a higher reported mortality than non-diabetic patients [23]. The second factor that was identified as a possible risk factor was the total cumulative dose of corticosteroids administered in the ICU. This is logical, as corticosteroids are a known suppressor of the immune system. However, because of lack of information on timing, no firm conclusions can be drawn on the temporal association between corticosteroid therapy and secondary infection. Routine administration of corticosteroids was not part of the Jessa protocol at the early stage of this study period and were only administrated according to clinical decision of the attending intensivist.

Common policy was to only start corticoids in patients with signs of refractory systemic inflammatory disease without diagnosed secondary infection. Thus, it cannot be ruled out that a third bystander, i.e. more severe COVID-19 disease or pre-existing comorbidities, may have independently increased both cumulative dose of corticoid therapy and risk of secondary infection. Nevertheless, current guidelines advise the administration of corticosteroids to COVID-19 patients treated in the ICU [24]. Apparently, any possible risk associated with the administration of corticosteroids is outweighed by its positive effect on the course of the infectious disease itself. Male gender was also associated with increased risk of secondary bacterial infection. Obviously, this association might also be elicited by a third bystander.

This study has several limitations. First, the retrospective single-center design and the small number of patients included in this cohort negatively impacts the generalizability of our findings. Second, due to the small sample, only univariable analyses could be performed. To confirm the described possible associations further research is necessary. Third, information on identification of bacteria on a species level was not always provided by the department of microbiology. Therefore, we chose to present only broad categories of bacteria (Gram-positive/negative bacilli/cocci).

## Conclusion

This study confirms that the incidence of secondary bacterial infections in critically ill patients infected with SARS-CoV-2 is very high. More specifically, these patients are at highest risk of developing secondary pneumonia, followed by bloodstream infection of unknown origin and catheter-related sepsis. Male gender, a history of diabetes mellitus and higher dosing of corticosteroids were associated with increased risk of secondary bacterial infection.

## Supplementary Information

**Additional file 1.** Standard treatment regimen of COVID-19 patients admitted to the ICU.

## Data Availability

Due to the applicable privacy regulation (GDPR) and Good Clinical Practices (GCP) legislation, the full underlying dataset supporting the study cannot be provided. This dataset contains potentially identifying information, for example age, BMI and comorbidities such as diabetes mellitus leading to a unique subject in the dataset. Therefore, descriptive statistics have been used for a general overview of our study population, and all other relevant information is provided in Table 1. Anonymized data is available on motivated request and can be send to: Prof. Dr. Björn Stessel; Stadsomvaart 11; 3500 Hasselt, Belgium; bjorn.stessel@jessazh.be; AND Jessa Ziekenhuis, Data Protection Officer (DPO); Stadsomvaart 11; 3500 Hasselt, Belgium; DPO@jessazh.be.
