# Supplementary material for: Secondary infection in COVID-19 critically ill patients: a retrospective single-center evaluation
Source: BMC Infect Dis. 2022 Mar 2;22:207. doi: 10.1186/s12879-022-07192-x (PMC8890021; doi:10.1186/s12879-022-07192-x)
Supplement: Supplementary file 1 — Additional file 1. Standard treatment regimen of COVID-19 patients admitted to the ICU. [file 12879_2022_7192_MOESM1_ESM.docx]

**Appendix**

**Infectious complications in COVID-19 critically ill patients: a retrospective single-center evaluation**

Astrid De Bruyn, MD^1^*; Stijn Verellen, MD^1^*; Liesbeth Bruckers, PhD^2^; Laurien Geebelen, MSc^1^; Ina Callebaut, PhD^1,2^; Ilse De Pauw, MD^1^; Björn Stessel, MD, PhD^1,3^; Jasperina Dubois, MD^1^

^1^ Department of Intensive Care and Anesthesiology, Jessa Hospital, Hasselt, Belgium

^2^ I-BioStat, Data Science Institute, Hasselt University, Martelarenlaan 42, 3500 Hasselt, Belgium

^3^ UHasselt, Faculty of Medicine and Life Sciences, LCRC, Agoralaan, 3590 Diepenbeek, Belgium

* joined first author

**Corresponding author:**

Jasperina Dubois

jasperina.dubois@jessazh.be

Dep. of Intensive Care and Anesthesiology

Jessa Hospital – Hasselt, Virga-Jesse Campus

Stadsomvaart 11

3500 Hasselt, Belgium

Phone +32 11 33 98 58

Fax +32 11 27 45 90

**Standard treatment regimen**

All COVID-19 patients were treated according to the COVID-protocol of the Jessa hospital, based on the latest insights on COVID-19 at a given moment in time (1). According to the protocol, all patients admitted to our ICU received an IV-infusion with glucose 5% as maintenance fluid and daily stress ulcer prophylaxis with Pantoprazole 40 mg intravenously. Prophylactic antibiotic therapy was initiated for 5 days, using Amoxicillin-clavulanic acid 1 g intravenously 4 times a day or Moxifloxacin 400 mg intravenously once a day in case of known allergy to penicillin. This prophylactic administration of antibiotics was abandoned as from 08^th^ April 2020. In addition, hydroxychloroquine was also routinely administrated: 400 mg orally the first day, followed by of 2 x 200 mg orally daily for 5 days, if no contra-indication was observed. However, also this regimen was rapidly abandoned, from May 2020, because of a lack of evidence of its beneficial effect. Sedation was performed routinely by a combination of propofol, midazolam and piritramide aiming for the lowest level of sedation required to tolerate mechanical ventilation. This regimen was sometimes completed with ketamine or dexmedetomidine if the initial schedule was inadequate. Ventilatory support was initiated with a high-flow nasal cannula or non-invasive mechanical ventilation as long as the patient was cooperative to this treatment. In case of respiratory fatigue, patients were sedated and intubated and invasive mechanical ventilation was started according to the ARDS-network guidelines. This was based on the first reports that the viral pneumonia caused by SARS-CoV-2 mimicked an ARDS-like pattern (1). At a later stage, very high PEEP levels, exceeding 15 mBar, were no longer administered. Therapy adjustments were guided by pulse oximetry-levels, which were continuously monitored and arterial blood gasses taken every 4 hours. In case of hypotension due to vasoplegia, norepinephrine was used as first choice vasopressor. All patients received routine low dose pharmacological VTE prophylaxis, i.e. once-daily subcutaneous injection of nadroparin calcium 2850 IU, according to the current guidelines in critically ill patients (2, 3). After the discovery of a relatively high incidence of deep venous thrombosis in our critically ill COVID-19-population on March 30^th^ 2020(4), we changed our prophylactic anticoagulation protocol from prophylactic to intermediate dosages of low molecular weight heparin (LMWH) with plasma anti-Xa activity monitoring (5). Anti-Xa activity was targeted at 0,3 to 0,5 IU/ml in patients without echographic findings of deep venous thrombosis (DVT) and 0,4 to 1 IU/ml in patients with screening duplex positive for DVT. Routine administration of corticosteroids was not part of the Jessa protocol at the early stage of this study period and were only administrated according to clinical decision of the attending intensivist. Based on the results of the RECOVERY-trial, patients were systematically treated with intravenous dexamethasone at a dose of 6mg once daily for up to 10 days starting from July 2020 (6).

1. Alhazzani W, Møller MH, Arabi YM, Loeb M, N. GM, Fan E, et al. Surviving Sepsis Campaign: guidelines on the management of critically ill adults with Coronavirus Disease 2019 (COVID-19). Intensive Care Medicine. 2020.

2. Kahn SR, Lim W, Dunn AS, Cushman M, Dentali F, Akl EA, et al. Prevention of VTE in nonsurgical patients: Antithrombotic Therapy and Prevention of Thrombosis, 9th ed: American College of Chest Physicians Evidence-Based Clinical Practice Guidelines. Chest. 2012;141(2 Suppl):e195S-e226S.

3. Schunemann HJ, Cushman M, Burnett AE, Kahn SR, Beyer-Westendorf J, Spencer FA, et al. American Society of Hematology 2018 guidelines for management of venous thromboembolism: prophylaxis for hospitalized and nonhospitalized medical patients. Blood advances. 2018;2(22):3198-225.

4. Pellens B, Romont M., Van Tornout M., De Mey N, Dubois J, De Pauw I, Ramaekers D, Stessel B. Prevalence of deep venous thrombosis in ventilated COVID-19 patients: a mono-center cross-sectional study Journal of Emergency and Critical Care Medicine 2020;4(31).

5. Stessel B, Vanvuchelen C, Bruckers L, Geebelen L, Callebaut I, Vandenbrande J, et al. Impact of implementation of an individualised thromboprophylaxis protocol in critically ill ICU patients with COVID-19: A longitudinal controlled before-after study. Thrombosis research. 2020;194:209-15.

6. Horby P, Lim WS, Emberson JR, Mafham M, Bell JL, Linsell L, et al. Dexamethasone in Hospitalized Patients with Covid-19. N Engl J Med. 2021;384(8):693-704.
